# Safety and efficacy of pulsed low-dose rate radiotherapy for local recurrent esophageal squamous cell carcinoma after radiotherapy: Study protocol for a prospective multi-center phase II trial

**DOI:** 10.1097/MD.0000000000016176

**Published:** 2019-06-28

**Authors:** Jie Li, Zhenhua Zhao, Guobo Du, Tangzhi Dai, Xuhai Zhen, Hongwei Cai, Dongbiao Liao, Miao Xiang, Yixue Wen, Lidan Geng, Xiyue Yang, Gang Feng, Yu Zhang, Jie Bai, Lei Liu, Xiaobo Du

**Affiliations:** a Department of Head and Neck Oncology, Cancer Center, and State Key Laboratory of Biotherapy, West China Hospital of Sichuan University, Chengdu, Sichuan; b Department of Oncology, Mian Yang Central Hospital, Mianyang; c Department of Oncology, Affiliated Hospital of North Sichuan Medical College, Nan Chong; d Department of Oncology, Lang Zhong People‘s Hospital, Lang Zhong; e Department of Rehabilitation Medicine, Mian Yang Central Hospital, Mianyang, China.

**Keywords:** esophageal squamous cell carcinoma, PLDR, re-irradiation

## Abstract

**Introduction:**

Re-irradiation after radiotherapy is a common treatment for locally recurrent esophageal cancer. However, the side effects of re-irradiation are serious. The most serious adverse reactions of re-irradiation include esophageal perforation and hemorrhage caused by esophageal perforation. Studies have shown that pulsed low-dose rate radiotherapy (PLDR) induces a hypersensitivity effect on tumor tissue and a hyper-repair effect on normal tissue, which can simultaneously reduce damage on the normal tissue and increase the therapeutic effect on the tumor. The objective of this study is to explore whether PLDR can reduce rate of esophageal perforation and improve efficacy in patients with recurrent esophageal squamous cell carcinoma (ESCC) after radiotherapy.

**Methods and analysis:**

This study is a prospective, multi-center, open, single-arm clinical trial designed to enroll 27 patients with locally recurrent ESCC after radiotherapy with or without chemotherapy. Re-irradiation will be performed using intensity modulated radiation therapy in 50 Gy/25 fractions. The strategy of PLDR includes dividing 2 Gy into 10 fractions, and administering each irradiating dose of 20 cGy at an interval of 3 minutes before the next low-dose irradiation. The actual dose rate of administration each time will be 16.67 cGy /minute. The primary endpoint in this study is the rate of esophageal perforation. The secondary endpoints are the objective remission rate, the palliative effect on quality of life and pain, and the time of disease progression. The observation time is 2 years after the end of the study.

**Trial registration:**

Clinical trial number: ChiCTR1900020609.

## 1 Introduction

Esophageal cancer (EC) is a common malignant tumor, and ranks seventh in incidence and sixth in mortality worldwide. According to 2018 statistics data, there have been 572,000 new cases of esophageal cancer and 509,000 deaths.^[1]^ Esophageal squamous cell carcinoma (ESCC) is the most common histological type globally, especially with a high incidence in developing countries.^[2]^ In China, EC ranks third in incidence and mortality, and more than 90% of the EC cases are reported with ESCC.^[3–4]^ Concurrent chemoradiotherapy is the standard treatment for locally advanced ESCC in patients who are resistant to or unsuitable for surgical treatment. About 50% of the ESCC cases are reported to recur locally within 3 years after chemoradiotherapy, and most recurrences occur in the target area of gross tumor volume (GTV).^[5,6]^ Most patients with recurrence die within 1 year without treatment. At present, there is no standard treatment for local recurrence after radiotherapy. Common treatment methods include surgery, re-irradiation, chemotherapy, and supportive treatment. The proportion of patients receiving esophagectomy after recurrence is very small. According to data from Anderson Cancer Center, definitive chemoradiation failed in 184 of the 276 patients treated, while only 23 (8%) underwent esophagectomy.^[7]^ In general, re-irradiation is superior to other treatments in patients who cannot be treated with esophagectomy.^[8–11]^ Re-irradiation is limited by the tolerance dose of normal tissues in the lungs, spinal cord, and esophagus and hence, increasing the target dosage is difficult. Re-irradiation can increase the risk of adverse reactions, such as esophageal perforation, hemorrhage, and radiation pneumonia. The most serious adverse reaction is esophageal perforation with an incidence of approximately 30%.^[9,12]^

**Figure 1:**
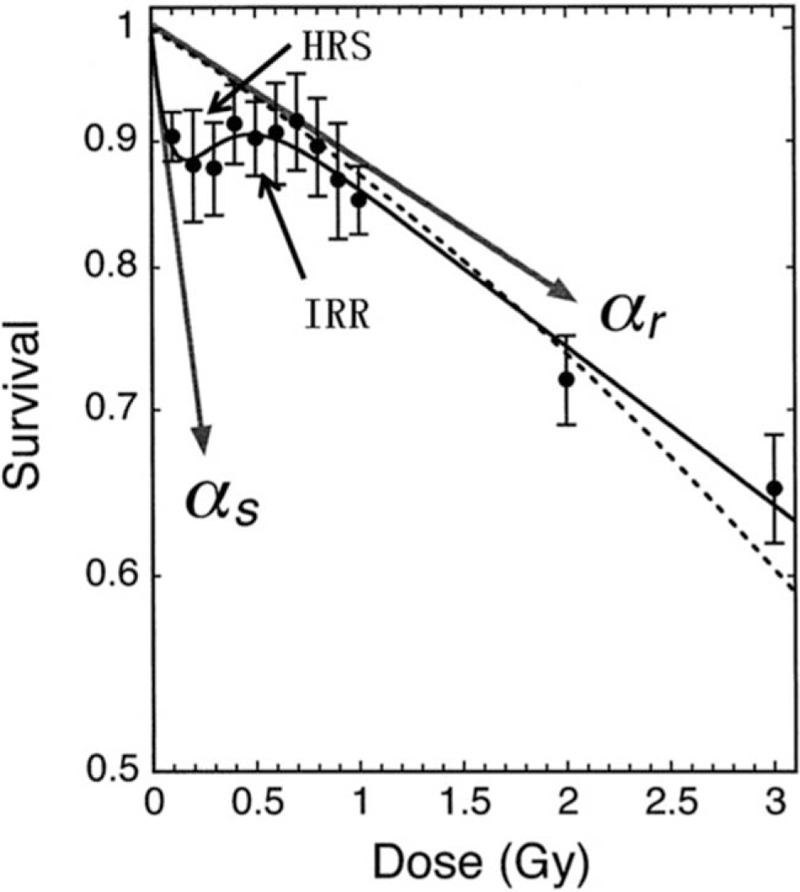
Classical cell survival curve of hypersensitivity. αr = the slope of survival curve of higher dose irradiation; IRR: irradiation resistance, αs = the slope of survival curve of low dose rate irradiation, HRS = Hypersensitivity.

Low-dose rate radiation is considered effective to alleviate the side effects of radiotherapy. According to the classical cell survival curve of hypersensitivity (Fig. 1),^[13]^ hypersensitivity (HRS) is observed in tumor cells exposed to <0.2 Gy low dose rate irradiation with a steeper slope of survival curve (αs) than that of higher dose irradiation(αr). When the dose reaches 0.2 to 0.8 Gy, radiosensitivity began to shift from sensitivity to irradiation resistance (IRR). An interval between each pulse exists during pulsed low-dose rate radiotherapy (PLDR). Sufficient interval time can promote repair of normal tissues and reduce side effects on normal tissues thereby increasing the therapeutic effect on tumors and reducing damage on normal tissues.

Researchers found that PLDR was more effective and exhibits lower toxicity to normal tissues than conventional radiotherapy in mouse models.^[14]^ The main reason for this is that PLDR causes less vascular damage, and retaining vascular network can improve tumor oxygenation. Normal tissue can be repaired during the treatment interval. Clinical studies of recurrent breast cancer, head and neck cancer, and glioma after radiotherapy have further demonstrated the effectiveness and safety of PLDR re-radiotherapy, and PLDR shows an increase in the ability to protect normal tissues.^[15–18]^ However, clinical studies of PLDR in patients with ESCC do not exist. Based on the effects of low dose radiosensitivity and low dose hyperrepair, we designed this study to explore whether intensity modulated radiation therapy (IMRT)-PLDR can reduce rate of esophageal perforation and improve efficacy in patients with recurrent esophageal squamous cell carcinoma (ESCC) after radiotherapy.

## 2 Methods/design

### 2.1 Recruitment and study design

**Figure 2:**
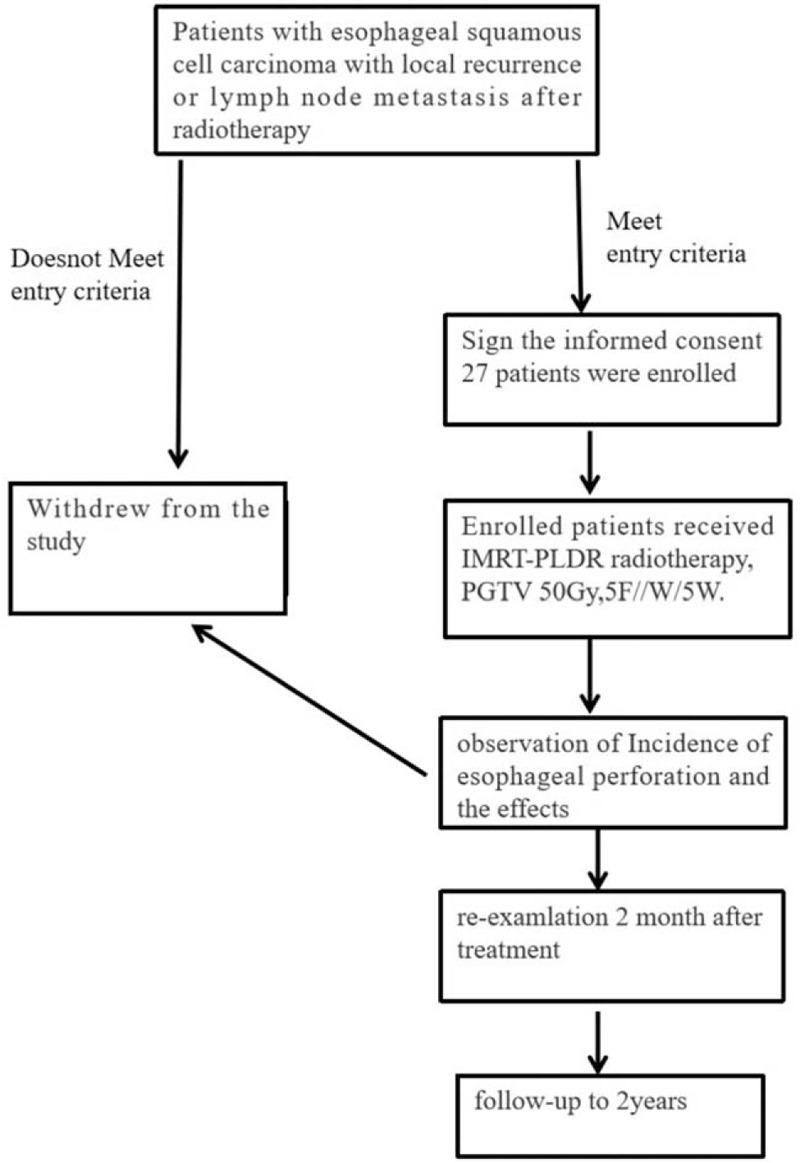
The clinical trial plan.

This study is a prospective, multi-center, open, single-arm phase II clinical trial designed to enroll 27 patients with locally recurrent ESCC after radiotherapy with or without chemotherapy (Fig. 2). Before the start of treatment, each eligible patient will be fully explained the ethics, purpose, design, implementation, requirements, and timing of the study.

### 2.2 Objectives

1. Primary endpoint
  Rate of esophageal perforation
2. Secondary endpoints
• Objective remission rate (ORR)
• Palliative effect on quality of life and pain
• Time of disease progression (TTP)

### 2.3 Inclusion criteria

1. Age, 18 to 75 years;
2. Pathological confirmation of ESCC;
3. Local recurrence and/or lymph node metastasis in the irradiation field of first radiotherapy and life expectancy of >3 months;
4. No distant metastasis;
5. Eastern Cooperative Oncology Group (ECOG) score of 0 to 2, which measures the patient's physical condition;
6. Patients who cannot tolerate surgery or the tumor cannot be removed surgically;
7. The patient is conscious and capable of coordination with positioning and treatment;
8. Availability of previous radiotherapy information, including total dose in the target area, segmentation mode, dose distribution, irradiation dose to normal tissue and isodose curve, can be obtained;
9. The patient has a measurable solid tumor (response evaluation criteria: RECIST 1.1); Women of childbearing age must be in a non-pregnancy state (pregnancy test negative), menopausal women with menopause for >12 months and considered infertile, women of childbearing age who have taken effective contraceptive measures to prevent pregnancy during the study period and 3 months after the end of the study;
10. Absolute neutrophil count (ANC) > 1.0 × 10^9^/L; Platelet (PLT) count – 75 × 10^9^ or higher/L;
11. Patients must sign a written informed consent and be willing to participate in and complete the study and follow up.

### 2.4 Exclusion criteria

1. patients with other malignant tumors;
2. patients who received radiotherapy for the target area within 4 weeks before entering this study;
3. patients with previous history of ataxia due to telangiectasia or other radiosensitivity reactions;
4. patients with scleroderma or active connective tissue disease;
5. patients with serious and uncontrollable infectious diseases;
6. Patients with severe mental or psychological disorders, central nervous system disorders, cardiovascular and cerebrovascular complications, epilepsy and other diseases which may limit their understanding, implementation, and treatment compliance of informed consent.

### 2.5 Subject withdrawal criteria

Criteria that will lead to the drop-out of a patient before completion of this study may be treatments for medical reasons or impairment that will result in an interruption or early completion of treatment, including:

1. Patient safety events;
2. Any other physical condition that researchers suspect to increase the risk of radiation-related toxicity;
3. Subjects voluntarily withdraw informed consent and request withdrawal from the clinical trial;
4. Disease progression with medical imaging evidence (such as CT, MRI, B-mode ultrasonography, etc.) or clinical progress judged by researchers (the cause of progress needs to be recorded in detail);
5. Pregnancy events during the study;
6. Other researchers consider it necessary to withdraw from the study (the reasons for withdrawal need to be documented in detail);
7. Patient participated in other clinical studies during this study;
8. Inadequate patient compliance.

### 2.6 Radiotherapy

**Figure 3:**
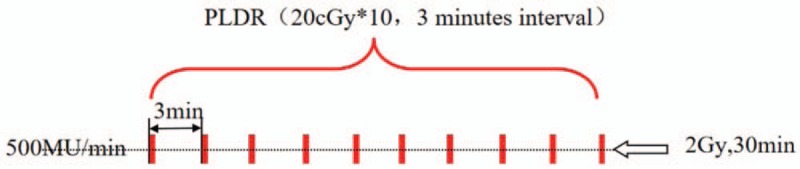
Schematic diagram of PLDR segmentation method.

Radiotherapy will be performed using IMRT in 50 Gy /25 fractions. The implementation strategy of PLDR include dividing 2 Gy into 10 fractions, and administering each irradiating dose of 20 cGy at an interval of 3 minutes before the next low-dose irradiation. The actual dose rate of administration each time will be 16.67 cGy/minutes (Fig. 3).

Target contour principle: GTV is defined as all by imaging examinations (esophageal barium meal, CT, esophageal lumen B to exceed, esophageal endoscopy, PET/CT, etc.) found in known generally tumor. GTV includes primary tumor (GTV-t) and enlarged metastatic lymph nodes (GTV-n). The definition of GTV-n is any of the following: the measured value of short diameter >1 cm during the diagnosis of CT scan or less than 1 cm, but met one of the following criteria: PET scan showed high metabolic absorption; lymph nodes fused or clustered; short diameter of tracheoesophageal groove lymph nodes >0.5 cm. In this study, all the enrolled patients were not exposed to preventive irradiation, and there was no need to delineate the clinical tumor volume (CTV). The planned tumor volume (PTV) includes the positioning error due to tissue and organ movement, and PTV= GTV + 0.5 cm. Actual output data can be determined from the quality control data of each center.

### 2.7 Data collection and management

A case report by researchers will accompany each case. Mianyang Central Hospital will be responsible for establishing the database and data entry, and will use a data acquisition system and an electronic data management system to manage data. Main researchers will audit the data and will lock it. The final version of the statistical plan will be completed and will be used to analyze the locked data. When the month and the year of treatment are clearly available, but the day is unknown, the date will be recorded as the fifteenth day of the month. When the year is clear, but the day and the month are unknown, the date will be recorded as the intermediate point between the end of the year and the last known date.

### 2.8 Assessment of the primary and secondary endpoints

Rate of esophageal perforation is the primary endpoint in this study, which will be observed from the initiation of re-irradiation to 60 days after the end of the study. Esophageal perforation should be diagnosed by esophageoscope. ORR is one of the secondary endpoints in our study. Ninety days after the end of treatment, we will evaluate the efficacy by using the RESICT 1.1 criterion through CT scan and esophageoscope. Quality of life scale QLQ-C30 (V3.0) according to the European Organization for Cancer Research and Treatment (EORTC) and digital pain scale will be assessed to determine the palliative effect on quality of life and pain in 2 years after the end of the study. Kaplan Meier curves will be performed to evaluate TTP, 2 years after the end of the study.

### 2.9 Statistical analysis

The power of the test is 80% and α = 0.05. According to the literature, the incidence of esophageal cancer perforation after re-irradiation is 30%. It is expected that after re-irradiation with PLDR technology, the rate of esophageal perforation will decrease to 10%. The optimal sample size for re-irradiation is 27 cases by checking the table (β = 0.20, α = 0.05).^[19]^ The proportion of patients with clinical response and the 95% confidence intervals will be calculated and reported. The Chi-Squared test and Kaplan–Meier method will be used to analyze the rates and severity of disease progression according to NCI-CTCAE (version 4), and Fisher exact probability test will be used to analyze the correlations between clinical outcomes and toxicity.

### 2.10 A quality assurance

IMRT-PLDR QA group will be established with 2 radiation oncologists and 1 physicist.

### 2.11 Ethics and dissemination

This trial was approved by the Ethics Committee of Mianyang Central Hospital, Sichuan Province, China with trial number: S2018071. The trial is subject to the supervision and management of the ethics committee. Written informed consent is obtained from all participants.

### 2.12 Trial status

The clinical registration was approved on January 10, 2019, This study opened to recruitment in February 2019, with a planned recruitment period of 1 year.

## 3 Discussion

Currently, there is no standard treatment available for local recurrence after radiotherapy. Numerous basic research studies reveal low-dose rate radiation to be effective to alleviate the side effects of radiotherapy. Based on the effects of low dose radiosensitivity and low dose hyperrepair, we designed IMRT-PLDR radiotherapy for local recurrence of ESCC after radiotherapy.

This study is novel in testing the safety and efficacy of PLDR for the treatment of locally recurrent ESCC. Our expected results are that PLDR causes fewer side effects and offers effective treatment, when compared with the historical data of conventional radiotherapy. The study results will indicate more treatment options for locally recurrent ESCC.

## Author contributions

Study conception LL and XBD. Initial Study design: XBD, LL and JL.

Revision of study design and protocol: XBD, LL,TZD and JL. Study coordination:

MX, YXW, LDG, XYY, GF, and YZ. Participating centers: Department of Oncology, Affiliated Hospital of North Sichuan Medical College, Nan Chong and Department of Oncology, Lang Zhong People‘s Hospital, Lang Zhong, China. Provide subjects for this study: ZHZ, GBD, XHZ, HWC, DBL. Drafting the manuscript:XBD, JL and JB. All authors read and approved the final manuscript.

**Conceptualization:** Jie Li, Lei Liu, Xiaobo Du.

**Data curation:** Zhenhua Zhao, Guobo Du, Tangzhi Dai, Xuhai Zhen, Hongwei Cai, Dongbiao Liao.

**Investigation:** Jie Li, Zhenhua Zhao, Guobo Du, Tangzhi Dai, Hongwei Cai, Dongbiao Liao, Miao Xiang, Yixue Wen, Lidan Geng, Xiyue Yang, Gang Feng, Yu Zhang.

**Methodology:** Jie Li, Zhenhua Zhao, Guobo Du, Tangzhi Dai, Xuhai Zhen, Dongbiao Liao, Miao Xiang, Yixue Wen, Lidan Geng, Xiyue Yang, Gang Feng, Yu Zhang.

**Project administration:** Xiaobo Du.

**Resources:** Xiaobo Du.

**Writing – original draft:** Jie Li, Xiaobo Du.

**Writing – review & editing:** Jie Bai, Lei Liu, Xiaobo Du.
